# The Preparation of Silver and Gold Nanoparticles in Hyaluronic Acid and the Influence of Low-Pressure Plasma Treatment on Their Physicochemical and Microbiological Properties

**DOI:** 10.3390/ijms242417285

**Published:** 2023-12-09

**Authors:** Armen Hovhannisyan, Magdalena Janik, Liliana Woszczak, Gohar Khachatryan, Magdalena Krystyjan, Anna Lenart-Boroń, Klaudia Stankiewicz, Natalia Czernecka, Dorota Duraczyńska, Zdzisław Oszczęda, Karen Khachatryan

**Affiliations:** 1 Scientific Technological Center of Organic and Pharmaceutical Chemistry of the National Academy of Sciences of the Republic of Armenia, Yerevan 0014, Armenia; armenarami@gmail.com; 2 Laboratory of Nanomaterials and Nanotechnology, Faculty of Food Technology, University of Agriculture, Balicka Street 122, 30-149 Krakow, Poland; magdalena.janik@student.urk.edu.pl (M.J.); liliana.woszczak@urk.edu.pl (L.W.); 3 Food Quality Analysis and Assessment, Faculty of Food Technology, University of Agriculture, Balicka Street 122, 30-149 Krakow, Poland; gohar.khachatryan@urk.edu.pl; 4 Department of Carbohydrates Technology and Cereal Processing, Faculty of Food Technology, University of Agriculture, Balicka Street 122, 30-149 Krakow, Poland; magdalena.krystyjan@urk.edu.pl; 5 Department of Microbiology and Biomonitoring, Faculty of Agriculture and Economics, University of Agriculture in Krakow, 30-059 Krakow, Poland; anna.lenart-boron@urk.edu.pl (A.L.-B.); klaudia.kulik@student.urk.edu.pl (K.S.); 6 Scientific Circle of Biotechnologists, Faculty of Biotechnology and Horticulture, University of Agriculture in Kraków, 29 Listopada Ave. 54, 31-425 Krakow, Poland; natalia.czernecka2@student.urk.edu.pl; 7 Jerzy Haber Institute of Catalysis and Surface Chemistry, Polish Academy of Sciences, ul. Niezapominajek 8, 30-239 Krakow, Poland; dorota.duraczynska@ikifp.edu.pl; 8 Nantes Nanotechnological Systems, Dolnych Młynów Street 24, 59-700 Bolesławiec, Poland; z.oszczeda@nantes.pl

**Keywords:** nanometals, nanosilver, nanogold, composites, antimicrobial activity, plasma water, hyaluronic acid

## Abstract

Nanometals constitute a rapidly growing area of research within nanotechnology. Nanosilver and nanogold exhibit significant antimicrobial, antifungal, antiviral, anti-inflammatory, anti-angiogenic, and anticancer properties. The size and shape of nanoparticles are critical for determining their antimicrobial activity. In this study, silver and gold nanoparticles were synthesized within a hyaluronic acid matrix utilizing distilled water and distilled water treated with low-pressure, low-temperature glow plasma in an environment of air and argon. Electron microscopy, UV-Vis and FTIR spectra, water, and mechanical measurements were conducted to investigate the properties of nanometallic composites. This study also examined their microbiological properties. This study demonstrated that the properties of the composites differed depending on the preparation conditions, encompassing physicochemical and microbiological properties. The application of plasma-treated water under both air and argon had a significant effect on the size and distribution of nanometals. Silver nanoparticles were obtained between the range of 5 to 25 nm, while gold nanoparticles varied between 10 to 35 nm. The results indicate that the conditions under which silver and gold nanoparticles are produced have a significant effect on their mechanical and antibacterial properties.

## 1. Introduction

Nanotechnology utilises the singular properties and behaviour of matter on a small scale, enabling the manipulation of material at the atomic level and paving the way for innovation in many areas of science and technology [1]. Due to its potential for significant breakthroughs, nanotechnology is an expanding field that has a vast impact on both our society and the future [2]. Nanotechnology has found diverse applications in several sectors of the economy, such as medicine, pharmaceuticals, transport, cosmetology, food, electronics, construction, and agriculture [3,4]. Its widespread use is attributed to its vast benefits and multifunctional capabilities, making it a crucial area of research and development for industries seeking to improve their products and processes.

Nanometals are a captivating area of research in nanotechnology. Various types of metal nanoparticles, such as iron, silver, gold, nickel, titanium, platinum, etc., have been developed and characterized by scientists [5]. The distinctive properties and potential applications of nanosilver and nanogold have prompted intensive research in numerous scientific fields. Nanosilver, also known as NanoAg, is highly valued for its potent antibacterial, fungicidal, antiviral, anti-inflammatory, anti-angiogenic, and anticancer properties. Consequently, silver nanoparticles (AgNPs) are extensively employed in medicine for various purposes such as dressings, bandages, antimicrobial creams, and disinfectants [6]. According to the literature, antimicrobial nanomaterials are often referred to as ‘nanoantibiotics’ and are regarded as one of the most promising methods for preventing or controlling the growth and spread of microbes and infections [7,8,9,10]. Nanosilver is highly chemically stable and resistant to corrosion, which allows it to maintain its properties even in highly acidic or alkaline environments [11]. Meanwhile, nanogold (nanoAu) can be adjusted to provide a variety of functions and applications. It can be functionalised by attaching a range of ligands, biological molecules, or polymers to the nanoparticle surface. Gold nanoparticles (AuNPs) have multiple applications as a drug carrier, imaging probe, catalyst, or sensor material [12,13,14]. Nanogold specifically exhibits amplified surface activity, chemical reactivity, and modified optical properties compared to larger gold particles [15]. Additionally, nanogold is highly biocompatible, which allows it to be well tolerated by living organisms [16]. It has broad applications in biomedical fields such as medical diagnosis [17], cancer treatment [18], drug transportation [19], and bioimaging [20,21].

The properties outlined create nanosilver and nanogold materials suitable for various applications. Consequently, one of the main areas of interest for nanotechnology researchers is the advancement of these nanometals in biopolymer carriers. Such research includes the development of active/intelligent packaging [22,23,24], dressings [25], and new formulations of cosmetics, including creams, shampoos, and conditioners [26,27,28]. Nanoparticles of metals embedded within polymer carriers are a captivating field of study, allowing for the advanced properties of metals at the nanoscale to merge with the biocompatible and environmentally sustainable nature of polymers. The polymers used as matrices for synthesising these nanoparticles can be categorised according to their origin: either as natural or synthetic polymers. Synthetic polymers, which are derived from non-renewable sources, cannot be naturally degraded and exacerbate environmental pollution [29]. Additionally, synthetic polymers may trigger an immune response and lead to allergic or toxic reactions within the body. By contrast, biopolymers—natural polymers—possess qualities such as biodegradability, renewability, and abundance in nature, making them a sought-after choice in multiple fields [30,31]. However, the production of nanowires in polysaccharide matrices poses several technological challenges. Regulating nanoparticle dimensions and morphology, maintaining stability, and ensuring uniform dispersion in the polysaccharide matrix are essential factors for acquiring nanostructures in polysaccharides. These components are crucial in the design of advanced materials incorporating nanostructures. Metal nanoparticles have undergone extensive investigations regarding their antimicrobial properties. The size and shape of these nanoparticles are paramount factors in determining their antimicrobial activity [32,33,34]. For instance, decreasing the size of metallic nanoparticles is predicted to enhance their antibacterial activity because of the notably larger surface area of the smaller nanoparticles [35]. Metal-based nanoparticles are known to have non-specific mechanisms of bacterial toxicity, which not only complicates the development of bacterial resistance, but also broadens the spectrum of antibacterial activity [36]. An antimicrobial assay employed fungi and bacteria, demonstrating the compounds’ stability, varying shape, size, and electronegative (capping) properties, culminating in enhanced antimicrobial functionality [37]. The size and shape of the metallic nanoparticles can be manipulated by altering the reducing agents, reaction conditions (including temperature and pH), environment, and carrier [34,38,39]. Various studies have investigated the impact of low-temperature plasma on the structure of water in the presence of different atmospheric gases such as air [40], nitrogen [41], methane [42], oxygen [43], ammonia [44], and carbon dioxide [45]. Additionally, the microbiological aspects of low-temperature plasma-treated water have also been explored [46,47,48,49]. Our previous investigation demonstrated that the application of plasma water impacted the polydispersity of nanoparticles and the optical characteristics of starch-based bionanocomposites [50]. Thus, the utilization of water treated with this method poses a promising research avenue.

Hyaluronic acid, among many natural polymers such as chitosan, sodium alginate, cellulose, and starch, is renowned for its exceptional chemical, physical, and biological properties. Hyaluronic acid is a foundational component found in all living organisms [51] and consists of the repeating disaccharides β-(1,4)-glucuronic acid and β-(1,3)-N-acetylglucosamine, which are repeatedly linked by alternating β-1,3- and β-1,4-glycosidic bonds [52]. In the human body, it forms several structures such as the extracellular matrix of connective tissues, blood vessel walls, cartilage, joint fluid, and the vitreous body of the eye. Moreover, it plays a role in the inflammatory response, angiogenesis, and tissue regeneration [53]. This polymer exhibits biocompatibility, biodegradability, and antimicrobial activity, which are additional advantages [54]. Additionally, the scientific literature suggests that hyaluronic acid with a higher molecular weight can demonstrate antioxidant activity, which in turn helps protect against free radicals [55]. Hyaluronic acid also offers excellent potential for modification due to its chemical structure containing various polymeric side groups such as hydroxyl, acetamide, and carboxyl groups [56]. As a result, this polymer provides an outstanding matrix for a variety of modifications, contributing to its overall versatility [57]. With the boundless potential of nanotechnology and the escalating desire for cutting-edge materials, bionanocomposites based on sodium hyaluronate and containing silver and gold nanometals demonstrate potential as innovative, biodegradable, and biocompatible substances for biomedical use [58].

Metal nanoparticles, including both silver and gold, were synthesised and characterised in a matrix composed of sodium hyaluronate polysaccharide in the context of this investigation. In this study, metallic nanoparticles were produced using methods consistent with the concept of ‘green chemistry’. Water was utilized as a solvent and treated with a low-temperature, low-pressure plasma in an atmosphere of air and argon. This enabled us to demonstrate the significant impact that even minor environmental modifications can have on the properties of the nanocomposites obtained (morphology, particle distribution, and physicochemical and microbiological properties). The findings yielded biodegradable nanocomposites that can rival synthetic materials in terms of properties, thus paving the way for a multitude of applications. This will greatly aid the cause of environmental conservation.

## 2. Results and Discussion

**Figure 1:**
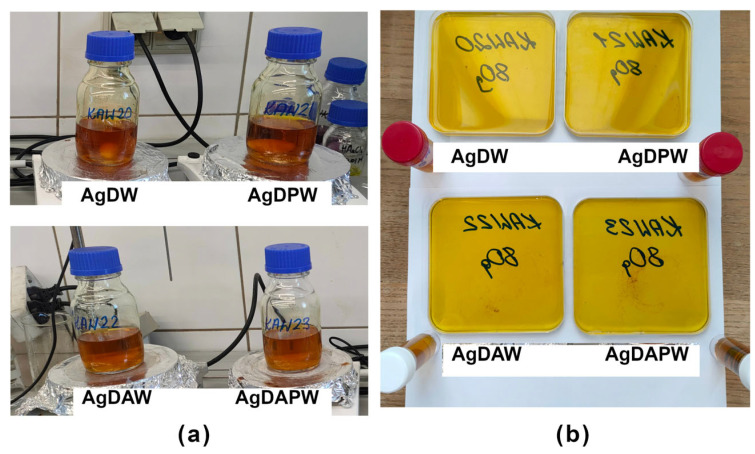
Hydrogels (**a**) and films (**b**) containing silver nanoparticles.

**Figure 2:**
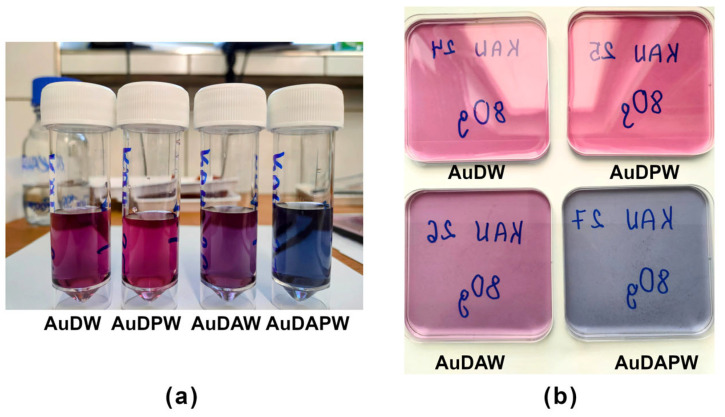
Hydrogels (**a**) and films (**b**) containing gold nanoparticles.

Figure 1a,b shows hydrogels and films containing silver nanoparticles. We can observe a slight difference in intensity and hue but no significant visual differences. This indicates that the reduction occurs in a very similar way regardless of the type of solvent (plasma-treated or not, air, or argon). Figure 2a,b, on the other hand, shows hydrogels and films for gold nanoparticles. In this case, we observe a clear difference in intensity and colour. This shows that the type of solvent plays an important role in shaping gold nanoparticles. Even with the naked eye, aggregates of gold particles can be seen in the AuDAPW sample (as in the resulting hydrogel as in the film).

**Figure 3:**
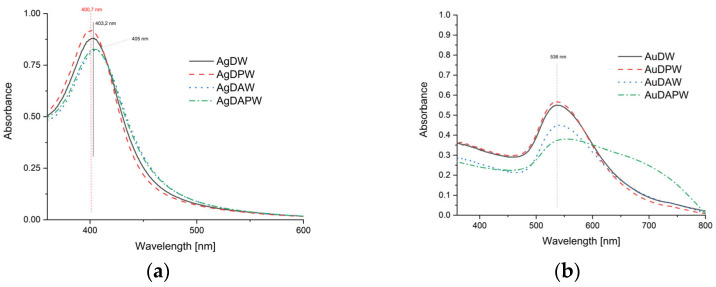
UV-Vis spectra of composites containing silver (**a**) and gold (**b**) nanoparticles.

To ascertain the shape and consistency of the nanoparticles produced, UV-Vis spectra were conducted on the resulting composites. Figure 3a displays the spectra for composites that contained silver nanoparticles. A distinctive absorption band was noticeable for silver nanoparticles at wavelengths of 400.7, 403.2 nm for AgDW and AgDPW composites, and 405 nm for AgDAW and AgDAPW. The use of plasma water had almost no impact on the size of the silver particles. Figure 3b displays the UV-Vis spectra representing the gold nanoparticles, which verified the original observations. An absorption band at approximately 536 nm was observed for the AuDW, AuDPW, and AuDAW samples, while a broad band in the 500–750 nm range denoted broad particle distribution and aggregation in the AuDAPW sample. Numerous studies [59,60,61,62,63] indicate the successful application of UV-Vis spectroscopy for detecting the existence of metallic nanoparticles. Furthermore, these studies demonstrate that the shape and position of the absorption band in the UV-Vis spectra of such particles are reliant on their size, shape, and distribution. It is therefore essential to consider these factors when analysing the spectra. Generally, an increase in the size of nanoparticles results in the shift of the absorption band towards longer wavelengths. Conversely, an increase in polydispersity is indicated by a broadening of the band. These findings partially align with our prior research [50], in which we identified the beneficial outcome of air-based plasma treatment on the shape and dimensions of the nanoparticles obtained. Nonetheless, for gold nanoparticles attained in water-based plasma under an argon atmosphere, there was a distinct rise in polydispersity, a definite increase in nanoparticle size, and aggregation. These findings were additionally validated through electron microscopy. Our research on the structure of plasma-treated water under argon, which is currently being prepared for submission, suggests the creation of argon/water clathrates that may affect nanoparticle aggregation.

The FTIR-ATR spectra in the spectral range of 750–4000 cm^−1^ for the films containing Hyal and AgNPs are shown in Figure 3. The vibrations ranging from 3600–2980 cm^−1^ are related to the N-acetyl side chain’s hydrogen-bonded O-H and N-H stretching vibrations. A set of overlapping bands with moderate intensity appear around 2910 cm^−1^, resulting from the C-H stretching vibrations. The stretching modes belonging to the planar carboxyl groups in hyaluronate are accountable for the 1620 and 1410 cm^−1^ bands, respectively, corresponding to asymmetric (C=O) and symmetric (C-O) vibrations [64]. We did not observe any significant changes in the spectrum of the composites compared to that of hyaluronic acid, which might have been due to the very low concentration of metallic nanoparticles.

**Figure 4:**
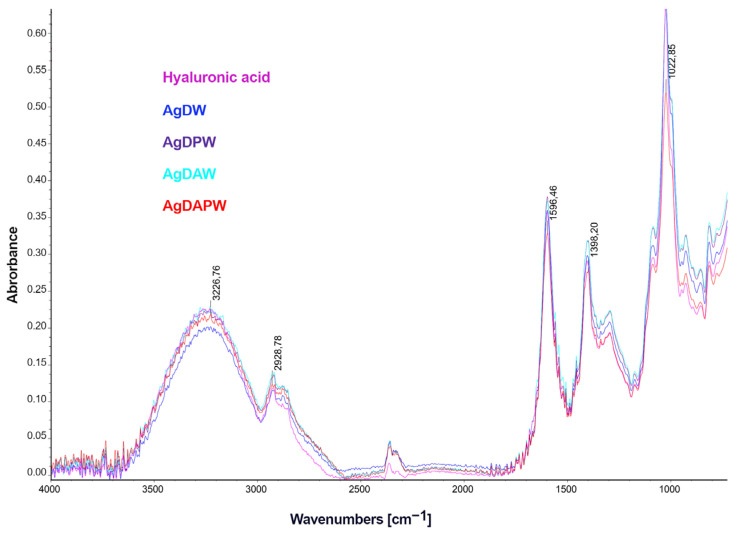
FTIR-ATR spectra of hyaluronic acid and composites containing silver nanoparticles.

Figure 4 illustrates the scanning electron micrographs of silver nanoparticles (AgDAW) (a) and gold nanoparticles (AuDAW) (b) that were recorded in backscattered mode to highlight chemical composition variation. A greater brightness contrast is indicative of higher atomic numbers or heavier elements.

**Figure 5:**
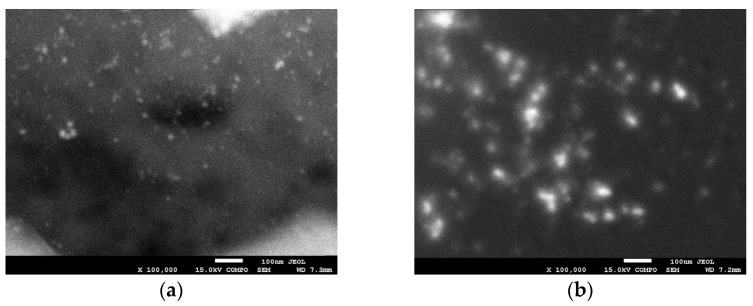
SEM images of AgDAW (**a**), AuDAW (**b**) recorded in backscattered electron (BSE) mode.

**Figure 6:**
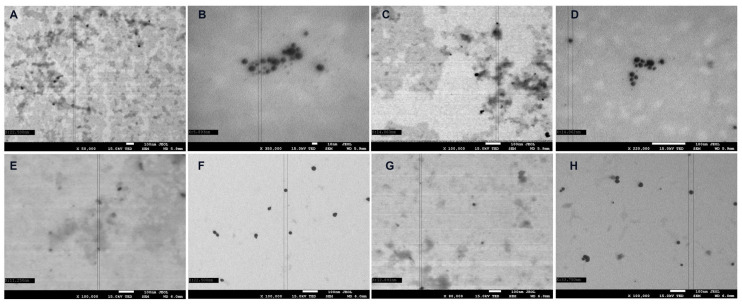
Electron microscopy images of AgDW (**A**), AgDPW (**B**), AgDAW (**C**), AgDAPW (**D**), AuDW (**E**), AuDPW (**F**), AuDAW (**G**), and AuDAPW (**H**) obtained with a transmission electron microscope detector.

**Figure 7:**
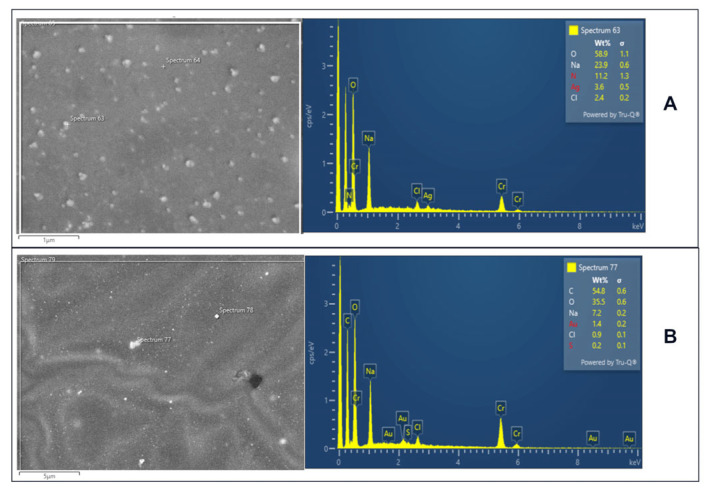
EDS analysis of the AgDPW (**A**) and AuDPW (**B**) composites.

The SEM (Figure 5) and TEM (Figure 6) analysis confirms our successful incorporation of both AuNPs and AgNPs within the hyaluronic acid matrix. This study provides evidence of uniformly distributed silver and gold nanoparticles of relatively uniform size on the hydrogel matrix, with AuNPs (varying from 10 to 35 nm) appearing slightly larger than AgNPs (5 to 25 nm). The size was significantly affected by the atmosphere during plasma treatment and by the use of plasma-treated water. Additionally, the presence of metallic NPs was confirmed through EDS analysis (Figure 7).

**Figure 8:**
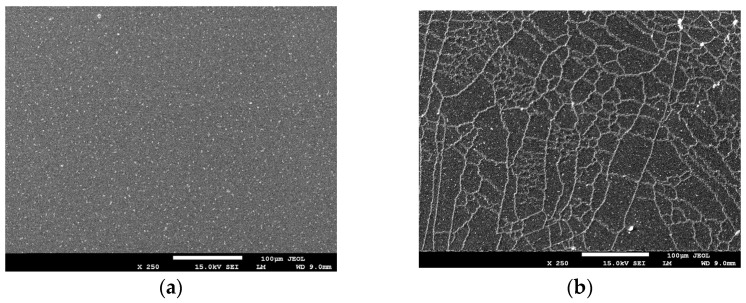
Comparison of low-magnification (LM) SEM images of ‘fresh’ AuDW (**a**) and AuDW exposed to high vacuum (**b**) for several hours.

We also observed that the polymer’s microarchitecture changed after residing for several hours in the specimen chamber under vacuum (9 × 10^−5^ Pa) (Figure 8). With time, chain-like structures emerged on the initially smooth surface, signifying alterations in the polymer structure due to a prolonged exposure to high vacuum.

This phenomenon is likely to be a result of the dehydration of the hydrogels, leading to the reorganisation of their molecular structure and consequent stiffening of the material [65,66].

The lowest water content was determined in AgDPW and AgDW composites, below 20%. In the other samples, the values exceeded 20% (Table 1).

Table 2 shows the opacity and colour parameters of the composite surfaces. Opacity is a characteristic that indicates the level of the light impermeability of a substance. The greater the value, the lower the transparency of the material and the higher its resistance to UV rays. The presented samples exhibited high opacity, and the nanocomposites with Ag were more resistant to UV than samples with Au. The transparency of the nanocomposites was unaffected by the type of water employed. Light-sensitive substances and foods require packaging with strong UV-blocking properties. The possibility of using the nanocomposites in packaging can have a positive effect on products with high light sensitivity. This assumption was confirmed by the colour parameters. The parameter L* of the tested nanocomposites was in the range of 37.84–61.66. The L* component describes the brightness of the colour from 0 to 100, where the maximum value indicates the brightest colour [67]. The values obtained testify to the significant darkness of the samples obtained. The nanocomposites with Au (AuDW, AuDPW, AuDAW, and AuDAPW) were characterized by higher brightness than composites with the addition of Ag (AgDW, AgDPW, AgDAW, and AgDAPW). In the case of the samples with AgNPs, the value of the a* component was positive (a* > 0), which indicated the dominance of the red colour. Samples with Ag were characterized by a significantly higher proportion of the red colour than those with Au, whose a* parameter indicated an equal proportion of red and green. The b* component represents the proportion of blue or yellow in the colour. Based on the data, it can be seen that AgNP samples were characterized by the predominance of yellow shades, as the values of this parameter were positive. The AuNP samples showed the opposite effect: the values of the b* parameter were negative, indicating a larger share of blue (b* < 0).

The thickness of the composites obtained and their mechanical properties are shown in Table 3. Based on the data, it can be seen that the film showed thickness in the range of 0.045–0.063 mm. Slight variations in thickness were observed between the composites despite the fact that the same amount of film-forming solution was poured onto the trays. This may have been due to the solid content enrichment in the samples [58,67]. There were visible effects of the type of metal presence in the matrix. Statistical analyses confirmed that the mechanical strength of nanocomposites containing Ag metal was on average 70% higher than nanocomposites containing AuNPs. The results indicate that the Ag nanocomposites obtained were mechanically stronger than the commonly used low density polyethylene (LDPE) films (16.5 MPa) and oriented polypropylene (OPP) (50.7 MPa), comparable with polyvinylidene chloride (PVCD) (65.6 MPa), but weaker than polyester (PE) (81.6 MPa) [68]. There were no statistically significant differences in the elongation at break between samples.

Although it is impossible to imagine the modern world without antibiotics, their excessive use in human and veterinary medicine, and in animal husbandry, has significantly contributed to the development and spread of drug resistance in microorganisms. Sublethal doses of antibiotics exert selective pressure on bacteria, promoting mutations and not allowing susceptible strains to survive and continue to transmit antimicrobial resistance [69]. This phenomenon significantly limits the possibilities and effectiveness of classic antibiotic therapy. The main animal pathogens contributing to the spread of drug resistance and posing a threat to humans include *Klebsiella*, *Proteus*, *Pseudomonas*, and *Staphylococcus aureus* (including methicillin-resistant *S. aureus*) [70]. Impaired wound healing associated with bacterial infections is common in both humans and animals. The formation of biofilms by pathogenic bacteria is the main factor impeding wound healing; it can cause inflammation and, in critical cases, can lead to the amputation of affected limbs or even death. Preventing biofilm formation by pathogenic bacteria is therefore of key therapeutic importance [71]. For the above reasons, new, effective, inexpensive, and non-toxic antimicrobial substances, which could be an alternative to antibiotics, are being sought. The antimicrobial potential of silver and gold nanoparticles, as well as plasma-activated water (PAW), have been observed [72,73,74]. In this experiment, we examined the antimicrobial efficacy of the composites incorporating silver and gold nanoparticles that were synthesised in various environments.

**Figure 9:**
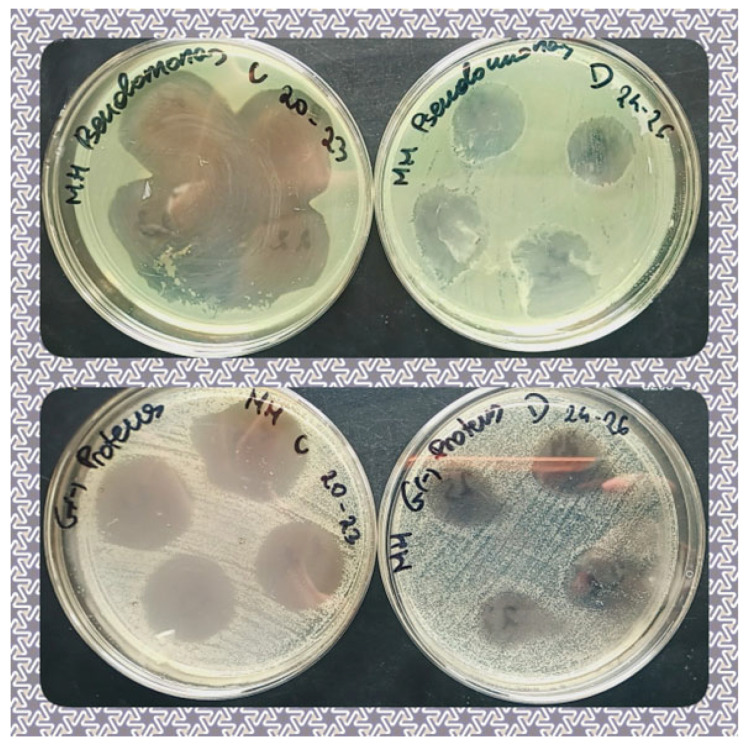
Growth inhibition caused by the AgNPs (AgDW, AgDPW, AgDAW, and AgDAPW marked, respectively, from 20 to 23) and AuNPs (AuDW, AuDPW, AuDAW, and AuDAPW marked, respectively, from 24 to 27), against *Pseudomonas* spp. (**top**) and *Proteus* spp. (**bottom**).

**Figure 10:**
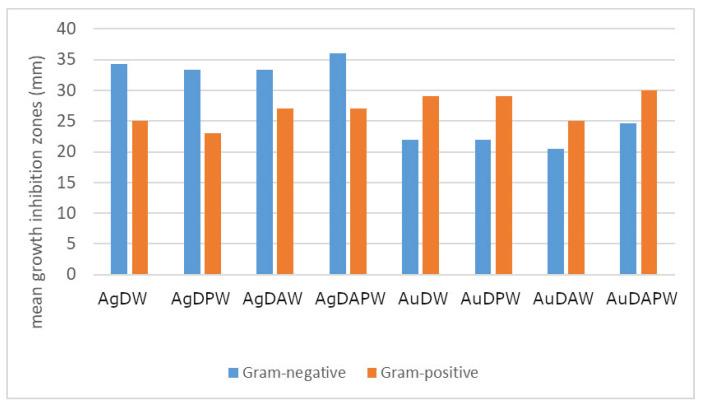
Mean growth inhibition zones (mm) caused by the application of films containing different experimental variants of AgNPs and AuNPs particles. The results are the means of the three replicates.

The antimicrobial activity of different foils was tested on four pathogenic and potentially pathogenic strains of bacteria isolated from the skin and wounds of humans and animals. The bacterial taxa included Gram-positive *S. aureus* and Gram-negative *Klebsiella*, *Proteus*, and *Pseudomonas* spp. The results of the readouts (means of triplicate measurements) are shown in Table 4. The growth of all the bacterial strains was inhibited by the experimental variants, and the statistical analysis showed that growth inhibition varied significantly between the examined variants and bacterial species/genera (*p* < 0.05). The strongest bactericidal properties, expressed as the largest growth inhibition zone diameter was observed for both AgDAPW and AuDAPW (plasma-treated distilled water saturated with argon; Table 4 and Figure 9). What can be seen in Table 4 and Figure 10 is that silver particles appeared to be more effective against Gram-negative *Klebsiella*, *Proteus*, and *Pseudomonas* than against *S. aureus*, and the effect was the opposite in the case of gold particles (i.e., growth inhibition caused by gold particles was stronger in the case of *S. aureus* than in the case of Gram-negative strains, Table 4 and Figure 10). Similarly, Ermolaeva et al. [72] examined the bactericidal effects of non-thermal argon plasma and observed that Gram-negative bacteria were more susceptible to plasma treatment than Gram-positives, suggesting cell wall thickness as one of the factors causing these differences. In general, the mechanisms of plasma-activated water (PAW) include the acidic condition, which plays a key role in the bactericidal effect of PAW, and high redox potential, which has been reported to disrupt the membrane integrity of microorganisms [75]. In their research on the impact of PAW on *E. coli*, Wang et al. [75] utilized a proteomic approach and found that the treatment led to a noteworthy increase in the oxidative stress defence protein and acid stress chaperone. PAW treatment led to a significant increase in the expression of conserved outer membrane lipoprotein, outer membrane porin protein, Omptin family outer membrane protease OmpT, and outer membrane protein X. This implies that PAW can cause damage to different membrane structures of *E. coli* [75]. The expression of the DNA repair protein, which is essential for the health and survival of organisms, was significantly down-regulated after PAW treatment. Also, phosphotransferase expression was down-regulated, resulting in reduced intracellular carbohydrate transport and phosphorylation, which may reduce the ability of *E. coli* to cope with nutritional stress or stress caused by physical and chemical factors. The varying effect of silver and gold nanoparticles against Gram-positive and Gram-negative bacteria has also been observed by, e.g., Elbehiry et al. [73] and Gouyau et al. [74]. Elbehiry et al. [73] examined the antibacterial effects and resistance induction of silver and gold nanoparticles in *S. aureus* associated with mastitis. Even though the antibacterial effects of AgNPs and AuNPs against *S. aureus* were comparable, AuNPs less frequently induced the resistance of this bacterium than AgNPs. Gouyau et al. [74] examined the potential antibacterial activity of 12 nm gold and silver nanoparticles against *S. aureus* and *E. coli* with very weak antibacterial results from gold nanoparticles against both species, no activity of AgNPs against *S. aureus*, and strong antibacterial activity against *E. coli*.

In summary, the combined measures of plasma-activated water saturated with argon both in AgDAPW and AuDAPW proved to have the most significant antibacterial effects. The effect varied between the silver and gold particles, with silver being more effective against Gram-negative bacteria and gold being more effective against Gram-positive *S. aureus*.

## 3. Materials and Methods

### 3.1. Materials

The following chemicals were utilized for producing the nanocomposites: high-molecular-weight hyaluronic acid (Aquajuv CT) with a molecular weight of 0.8–1.0 MDA; AgNO_3_ (Sigma-Aldrich, Poznań, Poland, 99.99%); HAuCl_4_·H_2_O (Sigma-Aldrich, 99.9%); D-(+)-xylose (Sigma-Aldrich, Poznań, Poland); and Argon Premier UN1006 (product code 16806) in the tank (100% purity) was purchased from Air Products Sp. z o.o. (Siewierz, Poland). None of the chemical reagents had been subjected to prior purification before being used in the experiments.

### 3.2. Methods

#### 3.2.1. Preparation of Plasma-Treated Water

Plasma water (DPW) was prepared following the method described in previous studies [40,41]. Distilled water (DW) (2000 mL) was placed in a glass volumetric flask, which was then positioned in the chamber of the reactor [41,76]. The solution was exposed to Glow Plasma for 30 min, where plasma of 38 °C was generated at 5 × 10^−3^ mbar, 600 V, 50 mA, and 280 GHz frequency. Plasma-treated argon-saturated distilled water (DAPW) was obtained via exposure to Glow Plasma for 30 min; argon-saturated distilled water (DAW) was obtained by passing a jet of argon at 10 litres per minute at 0.15 MPa for 2 h through 2000 mL of DAW.

The water produced was stored in sealed Teflon containers.

#### 3.2.2. Hyaluronic Acid Hydrogel Preparation

A 2% solution, weighing 200.0 g, was prepared by weighing 2.0 g of hyaluronic acid on an analytical balance (Radwag, Białystok, Poland) and adding 198.0 millilitres of distilled water. The mixture was stirred using a magnetic stirrer (Heidolph RZR 2020, Heidolph Instruments GmbH & Co. KG, Schwabach, Germany) until it formed a clear gel.

These steps were identically replicated with plasma-treated distilled water (DPW), argon-saturated distilled water (DAW), and argon-saturated distilled water that was subsequently treated with plasma (DAPW).

#### 3.2.3. Preparation of Ag and Au Nanoparticles

To the hyaluronic acid hydrogels (200.0 g) obtained in the previous section, we added 10.0 mL of AgNO_3_ solution (0.0100 M) and 6.0 mL of 4% xylose solution (as a reducing agent). We stirred the resulting mixture on a magnetic stirrer (Heidolph RZR 2020, Heidolph Instruments GmbH & Co. KG, Schwabach, Germany) for 5 h. The resulting nanosilver suspension in the hyaluronic acid matrix was then cooled to room temperature and applied to 120 × 120 mm plastic dishes in an amount of 80.0 g, before being left to dry. The resulting composites were stored in sealed dishes. Four samples were obtained depending on the type of water used. The samples were labelled accordingly: AgDW, AgDPW, AgDAW, and AgDAPW.

To the hyaluronic acid hydrogels (200.0 g) obtained in Section 3.2.2, we added 2.6 mL of HAuCl_4_ solution (0.0100 M) and 2.0 mL of 4% xylose solution (as a reducing agent). We stirred the resulting mixture on a magnetic stirrer (Heidolph RZR 2020, Heidolph Instruments GmbH & Co. KG, Schwabach, Germany) for 5 h. The resulting nanogold suspension in the hyaluronic acid matrix was then cooled to room temperature and applied to 120 × 120 mm plastic dishes in an amount of 80.0 g, before being left to dry. The resulting composites were stored in sealed dishes. Four samples were obtained depending on the type of water used. The samples were labelled accordingly: AuDW, AuDPW, AuDAW, and AuDAPW.

#### 3.2.4. Water Content

Water content was determined according to the Krystyjan et al. [77] procedure. The composites were cut into rectangular specimens (2 × 2 cm^2^) and weighed in an analytical balance to obtain the initial weight of the sample (M1). The specimens were then subjected to drying in an oven at 70 °C for 24 h, with the initial dry mass (M2) subsequently determined gravimetrically.

The water content of the composites was calculated according to Equation (1), as follows:

Water content (%) = (M1 − M2)/M1 × 100 (1)

#### 3.2.5. Opacity

The UV impermeability of the films was assessed by exposing rectangular film samples to 600 nm light absorption in a Helios-Gamma 100–240 UV/V spectrophotometer [77]. The test cell of the spectrophotometer directly held the film samples, while the empty cell acted as a reference. The opacity of the films was then ascertained by using Equation (2) as follows:

Opacity = A_600_/x (2)

where A_600_ represents the absorption at 600 nm and x is the thickness of the film expressed in millimetres; the analyses were conducted in five separate replicates.

#### 3.2.6. Surface Colour Measurements

Surface colour was measured using Konica MINOLTA CM-3500d equipment from Konica Minolta Inc. in Tokyo, Japan. A 30 mm diameter window was utilized with reference to the D65 illuminant/10° observer. The results were expressed using the CIELab system, with the following parameters determined: L* (L* = 0 black, L* = 100 white), a*—proportion of green colour (a* < 0) or red (a* > 0), and b*—proportion of blue (b* < 0) or yellow (b* > 0) [77]. The measurements were taken on a standard white background, and the experiment was repeated five times for accuracy.

#### 3.2.7. Thickness Measurement

Composite thickness was measured using a micrometre with a 0.001 mm resolution, catalogued under number 805.1301 (Sylvac SA, Crissier, Switzerland). The sample thickness was obtained as the average of five measurements taken from different locations within the gauge length area [78].

#### 3.2.8. Mechanical Properties of the Composites

The dried materials obtained were subjected to conditioning inside desiccators at 25 °C and 55% relative humidity (RH). The conditioning was performed by using magnesium nitrate-6-hydrate saturated solutions for 48 h before analysis. The samples were prepared according to standards [79] and were determined using the TA-XT plus texture analyser (Stable Micro Systems, Haslemere, UK). The films were then cut into strips of 35 × 6 mm^2^ and placed into holders. The holders were initially separated by 20 mm with a grip separation rate of 2 mm/min. To calculate the tensile strength (TS), the maximum force at the film’s rupture was divided by its cross-sectional area. Elongation at the break was expressed as a percentage (EAB) by dividing the elongation at rupture by the initial gauge length and multiplying by 100 [78]. The average values of 10 replications were reported.

#### 3.2.9. UV-Vis Absorption Spectrophotometry

The UV-Vis absorption spectra for the composites were obtained by utilizing a Shimadzu 2101 (Shimadzu, Kyoto, Japan) scanning spectrophotometer within the 300–800 nm range. The procedure involved situating the film fragments in a 10 mL quartz cuvette, with an empty cuvette employed as the reference point.

#### 3.2.10. FTIR-ATR Spectrophotometry

The FTIR-ATR spectra of the composites were analysed with a MATTSON 3000 spectrophotometer (Madison, WI, USA). The range analysed was from 4000 to 700 cm^−1^ with a resolution of 4 cm^−1^. The spectrophotometer was equipped with a 30SPEC 30 Degree Reflectance adaptor (MIRacle ATR, PIKE Technologies Inc., Madison, WI, USA).

#### 3.2.11. Scanning Electron Microscopy (SEM) and Energy Dispersive X-ray Spectroscopy (EDS)

Scanning electron microscopy (SEM) and energy dispersive X-ray spectroscopy (EDS) analysis were carried out with the aid of a JEOL JSM-7500F microscope (JEOL, Tokyo, Japan) coupled with an AZtecLiveLite Xplore 30 system (Oxford Instruments, Abingdon, UK). Before the analysis, the samples underwent a coating process involving a 20 nm layer of Cr using a K575X Turbo Sputter Coater (Emitech, Ashford, UK). A scanning electron microscope was equipped with a transmission electron microscope (TEM) detector. Samples for TEM analysis were prepared by drop-coating 10 µL of the sample on carbon-coated 200 mesh copper (100) grids (TAAB Laboratories, Aldermaston, Berkshire, UK).

#### 3.2.12. Isolation and Identification of Tested Microorganisms

Swab samples were taken from wounds and lesions on the skin and bodies of humans and animals. The samples were cultured on selective and chromogenic media to distinguish and identify bacterial strains of the commensal skin microbiota, pathogens, and potential pathogens. Columbia Agar with Sheep Blood Plus (Oxoid, Cheshire, UK) and Baird-Parker agar (Oxoid, Basingstoke, UK) were utilized to distinguish and identify *Staphylococcus aureus* (grey to black colonies with a clear halo on BP agar, white colonies with haemolysis on CA, incubated for 24–48 h at 37 ± 1 °C). UTI agar Plus (Oxoid, Cheshire, UK) was utilised to isolate and identify Gram-negative pathogens, including *Proteus, Klebsiella*, and *Pseudomonas* by incubating for 24–48 h at 37 ±1 °C. *Proteus* presented clear colonies with a brown halo, *Klebsiella* produced purple colonies, and *Pseudomonas* colonies were clear and colourless. After initial identification, the chosen bacterial colonies, consisting of Gram-positive (*Staphylococcus aureus*) and Gram-negative (*Klebsiella*, *Proteus*, and *Pseudomonas*) bacteria, were subcultured and utilized for the microscopic scrutiny of Gram-stained slides. Subsequently, the taxonomic position of the selected bacterial strains was validated using MAL-DI-TOF (matrix-assisted laser desorption/ionization-time of flight) mass spectrometry.

#### 3.2.13. Antimicrobial Activity of Tested Agents

Antimicrobial activity was assessed on four bacterial isolates, namely Gram-positive *S. aureus* and Gram-negative *Klebsiella, Proteus*, and *Pseudomonas*. Antimicrobial activity was assessed on four bacterial isolates, namely Gram-positive *S. aureus* and Gram-negative Klebsiella, Proteus, and Pseudomonas. The bacterial cultures were transferred into sterile saline solutions to obtain 0.5 MacFarland suspensions, which were then streaked onto Mueller–Hinton agar (Biomaxima, Lublin, Poland). Technical abbreviations were explained when first used. The foils were sterilised in UV light for 30 min. We cut 10 × 10 mm squares with surface-sterilised scissors and then applied them to the surface of bacterial cultures. Following an incubation period of 18–24 h at 37 ± 1 °C, the growth inhibition zones around the foil fragments were measured to obtain the results. As the applied foils were square, two diameters were read, and the final result was expressed as the mean of the two readings (mm). We conducted all the experiments in triplicate.

#### 3.2.14. Statistical Analysis

Statistica v.13 (TIBCO Software, Palo Alto, Santa Clara, CA, USA) was used to perform the statistical analysis. The growth inhibition descriptive statistics, including mean, standard deviation, and coefficient of variation, were calculated.

For the microbiological tests, a one-way ANOVA, followed by a post hoc Tukey’s test were applied to assess the significance of differences in the growth inhibition between the applied experimental variants and between the reaction of bacterial species. The significance level for all tests was predetermined as *p* < 0.05.

For water content, mechanical properties, and surface colour analysis, the one-way analysis of variance (ANOVA) Fisher test was carried out (*p* ˂ 0.05).

## 4. Conclusions

This study shows that the type of solvent did not play a significant role in the formation of silver nanoparticles, while it had a significant effect on the formation of gold nanoparticles. SEM/TEM analysis confirmed the successful incorporation of both AuNPs and AgNPs into the hyaluronic acid matrix. This study demonstrated that the application of plasma-treated water had a significant effect on the size and distribution of metal nanoparticles. Silver nanoparticles ranging from 5 to 25 nm were obtained, while the size of gold nanoparticles varied from 10 to 35 nm. The electron microscopy observations correlated with the UV-Vis spectroscopy results.

We did not observe any significant changes in the FTIR-ATR spectra of the composites compared to that of hyaluronic acid, indicating that the generation of metallic nanoparticles had no significant effect on the chemical structure of the biopolymer.

The presence of AgNPs significantly improved the mechanical properties of the bionanocomposites obtained compared to commonly used synthetic polymers.

All the obtained biocomposites containing silver and gold nanoparticles exhibited antimicrobial properties. Among all the composites tested, films containing AgNPs and AuNPs generated in plasma-treated water in an argon atmosphere stood out. AgNPs were more effective against Gram-negative bacteria, and samples containing AuNPs were more effective against Gram-positive S. *aureus*.

Plasma-treated water, saturated with various gases, can produce materials with diverse properties, unaffected by chemical compounds or alterations to production processes and equipment.

## Figures and Tables

**Table 1:** Water content in composites.

Sample	Water Content (%)
AgDW	17.62 ± 0.33 ^d^
AgDPW	15.22 ± 0.57 ^e^
AgDAW	21.27 ± 0.08 ^c^
AgDAPW	21.43 ± 0.00 ^c^
AuDW	24.96 ± 1.36 ^b^
AuDPW	21.54 ± 0.47 ^c^
AuDAW	20.90 ± 0.44 ^c^
AuDAPW	27.98 ± 1.49 ^a^

Values are expressed as mean ± SD. The same superscript letters in each column demonstrate a lack of significant difference between values (*p* ˂ 0.05).

**Table 2:** Colour of the surface of the films.

Sample	Opacity	L* (D65)	a* (D65)
AgDW	12.29 ± 0.10 ^a^	37.84 ± 0.93 ^g^	9.13 ± 0.39 ^d^
AgDPW	11.17 ± 0.77 ^b^	44.17 ± 0.89 ^e^	16.26 ± 1.08 ^b^
AgDAW	12.04 ± 0.33 ^a^	43.24 ± 0.74 ^f^	13.96 ± 1.02 ^c^
AgDAPW	12.02 ± 0.19 ^a^	43.54 ± 0.45 ^e,f^	17.20 ± 0.45 ^a^
AuDW	7.96 ± 0.31 ^c^	59.65 ± 0.99 ^b^	1.83 ± 0.07 ^g^
AuDPW	7.30 ± 0.24 ^d^	53.32 ± 0.93 ^d^	3.57 ± 0.43 ^f^
AuDAW	6.92 ± 0.15 ^d,e^	61.66 ± 0.71 ^a^	−0.13 ± 0.05 ^h^
AuDAPW	6.48 ± 0.10 ^e^	56.12 ± 0.48 ^c^	6.85 ± 0.53 ^e^

Values are expressed as mean ± SD. The same superscript letters in each column demonstrate a lack of significant difference between values (*p* ˂ 0.05).

**Table 3:** Mechanical properties of the films obtained at 25 °C and 55% humidity.

Sample	Thickness (mm)	TS (MPa)	EAB (%)
AgDW	0.062 ± 0.014 ^a,b^	59.15 ± 8.46 ^c^	3.67 ± 0.90 ^a^
AgDPW	0.063 ± 0.006 ^a^	74.05 ± 8.98 ^a^	2.87 ± 0.33 ^b^
AgDAW	0.047 ± 0.004 ^d,e^	69.53 ± 7.08 ^a,b^	2.89 ± 0.39 ^b^
AgDAPW	0.045 ± 0.004 ^e^	66.93 ± 4.83 ^b^	3.22 ± 0.47 ^a,b^
AuDW	0.061 ± 0.010 ^a,b^	46.48 ± 6.25 ^d^	3.33 ± 0.21 ^a,b^
AuDPW	0.058 ± 0.007 ^a,b,c^	36.27 ± 2.81 ^e^	3.45 ± 0.17 ^a,b^
AuDAW	0.052 ± 0.003 ^c,d^	30.65 ± 2.71 ^e^	3.64 ± 0.22 ^a^
AuDAPW	0.054 ± 0.008 ^b,c,d^	45.08 ± 3.34 ^d^	3.17 ± 0.58 ^a,b^

Values are expressed as mean ± SD. The same superscript letters in each column demonstrate a lack of significant difference between values (*p* ˂ 0.05). TS—tensile strength; EAB—percent elongation at break.

**Table 4:** Growth inhibition zones (mm) for bacterial strains as a result of the application of composites containing Ag and Au NPs.

Genus/Species Sample	*Klebsiella*	*Proteus*	*Pseudomonas*	*S. aureus*
AgNPs				
AgDW	28	37	38	25
AgDPW	29	36	35	23
AgDAW	28	37	35	27
AgDAPW	30	38	40	27
mean	37	28.75	37	25.5
standard dev.	2.45	0.96	0.82	1.91
CV (%)	7	3	2	8
AuNPs				
AuDW	25	15	26	29
AuDPW	25	14	27	29
AuDAW	22	12	22	25
AuDAPW	28	16	30	30
mean	26.25	25.25	14.25	28.25
standard dev.	3.30	2.06	1.71	2.22
CV (%)	13	8	12	8

## Data Availability

The data presented in this study are available on request from the corresponding author.
